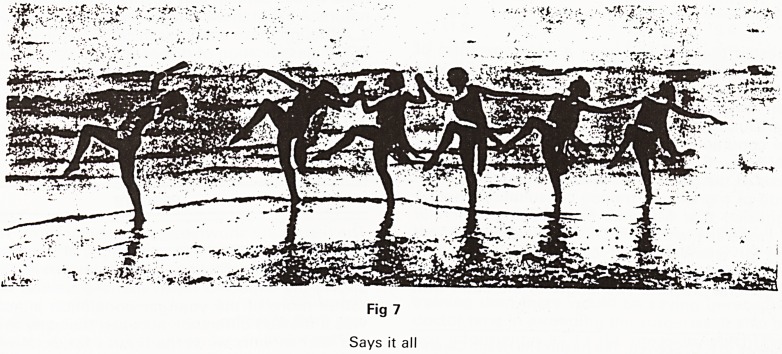# Domestic Medicines. An Irreverent Look at Health Education

**Published:** 1988-08

**Authors:** Martin Crosfill

**Affiliations:** Consultant Surgeon, W. Cornwall Hospital, Penzance


Bristol Medico-Chirurgical Journal Volume 103 (iii) August 1988

### Domestic Medicines
### An Irreverent Look at Health Education

**Martin Crosfill F.R.C.S.**
Consultant Surgeon, W. Cornwall Hospital, Penzance.

*Rise early in the mourne and straight remember*
*With water cold to wash your hands and eyes,*
*In gentle fashion retching every member,*
*And to refresh your braine, when, as you rise*
*In heat, in cold, in July and December*
*Both combe your head and brush your teeth likewise*

The thirteenth century Regimen Sanitatis of Salerno, quoted here in a translation by Sir John Harington was merely the forerunner of many books on the maintenance of health. Such books, written by doctors for members of the general public give, I submit, a more accurate view of the state of medical belief and practise than do many that are written by doctors for doctors; they deserve to be subjected to scholarly analysis. Unfortunately they are too diverting.

Roughly contemporaneous with Harington's translation were books such as the Garden of Health, the Castell of Health, the Breviary of Health and the Haven of Health. The latter, by Thomas Coghan is addressed to students and gives detailed instructions on how to occupy the twenty four hours.

Students should:

> *"Apply themselves earnestly to reading and meditation for the space of an hour and then remit a little in their cogitation and meantime with a combe to combe their head from before backwards about forty times and to rub their teeth with a coarse linen cloth then to return again to meditation for two hours."*

Medical thought in the sixteenth century was still dominated by the humoral theories of Hippocrates and Galen, yet it is interesting to see how the systematic listing of clinical signs and symptoms begins to surface.

> *"The Hearte sick: difficulties of breath, trembling of the hart, beating of the pulse, fever, colde, diversities of colours, griefe about the herte*.*

Some of the ideas have a peculiarly modern ring:

> *"Brown bread, made of the coarsest of wheat flour, having in it much branne, filleth the belly with excrements ... such as have been used to fine bread, when they have been costive, by eating brown bread and butter, have been made soluble."*

If we are to take this advice, let us not forget Sir John Harington's warning:

> *"Great harmes have growne, and maladies exceeding by keeping in a little blast of wind ..."*

As we pass from the sixteenth to the seventeenth century the emphasis shifts from health to disease and from exhortation to fact. Publications take a more encyclopaedic form, typical of which is Culpeper's "Family Physician". This book contains anatomical and clinical sections and a dispensatory. The anatomical descriptions are detailed and interesting; what can, from the illustration, only be the adrenals are described quaintly as 'deputy kidneys' but also as 'black choler cases with an apparent internal cavity furnished with a dreggy and black humour' .... surely the description of a patient who died of fulminating toxaemia. The writing becomes more detailed as one nears the pubis and almost as much space is devoted to the penis and scrotum as there is to the thorax. The high moral tone adopted in the clinical section together with the salacious anatomical detail must have done wonders for the sales.

**Fig 1**

The G.U. system from Culpeper's Family Physician

There were, of course, plenty of books specifically for women but space permits mention of only two. A curious little book, "The Works of Aristotle the Great Philosopher" was first published in 1684 and remained in print as a potent source of worry and misinformation for nearly two hundred years. Many a mother-to-be (having dutifully lain on her right side after copulation in order to produce a male child) must have fretted lest unforeseen prenatal events might lead her to produce a monster. Several of these, including Siamese twins and a phocomelic are graphically illustrated, as in the Ravenna Monster. This picture well antedates the publication having been 'borrowed' from John Sadler (The Sick Woman's Private Looking Glasse, 1636). He in turn borrowed it from Ambroise Paré who was quoting from even earlier sources. The whole of the first section of the

* Sir Thomas Elyot Castell of Health (1534). He had a cavalier attitude to spelling.

**Fig 2**

The Ravenna monster book, the 'Masterpiece' is medieval in tone both in its repeated reference to classical authors and to scriptures and in its remedies; thus for menorrhagia –

> *"Asses dung is approved of whether taken inwardly with quinces or outwardly ... let her take one scruple and a half of pilon in water, make a suffumagation for the matrix (with) frankincense, burnt frogs, not forgetting the hoof of a mule ..."*

Despite this the second half of the book contains quite sensible directions for the midwife; it was presumably added later but with minimal revision of the original material.

The other book, taken out of its chronological sequence, is "Advice to a Wife" published about 1814 by Henry Chevasse, FRCS. It provides ammunition for the feminist lobby from the very first sentence:

> *"A good wife is Heaven's best gift to man."*

He continues:

> *"How often a lady is, during the first year of her wifehood, gadding out night after night, one evening to a dinner party, the next night to private theatricals, the third to an evening party, the fourth to the theatre, the fifth to a ball, the sixth to a concert ..."*

(one can see the class of readership he was aiming at)

> *"Fashion is oftentimes but another name for suicide and baby slaughter ...... a young married lady ought at once to commence taking regular and systematic outdoor exercise which might be done without in the least interfering with her household duties ..."*

The poor girl who has been recommended in the sacred name of ventilation to turn her house into a cross between a wind tunnel and a refrigerator is next told how to wash herself:

> *"A young wife ought to strip to the waist and then proceed to wash her face after the following manner; she should fill the basin three parts full with rain water then, having well soaped and cleansed her hands she should re-soap and dip her face in the water ..."*

and so on for six pages of detailed instructions. Not all attributes are as important as cleanliness:

> *"A husband soon becomes tired of great performances on the piano, of crochet and worsted works and other fiddle faddle employment, but he can always appreciate a good dinner ..."*

One has a feeling that something was missing from Chevasses's life – let us hope he found a good cook. He could probably afford one, his book, thirty years after publication, was still selling over ten thousand copies annually.

If we return now to the eighteenth century we find we have just emerged from a world of humours and influences into what is, for us, a more rational one where specific diseases have specific causes and, by implication, specific remedies. Domestic medicines reflect this change. Probably the most successful book of its kind was Dr William Buchan's "Treatise on the Prevention and cure of Diseases" (1769).

Buchan starts with some general directions for healthy living and his style is direct and appealing. On the subject of swaddling, for instance, he says:

> *"It would be a difficult task to persuade the generality of mankind that the shape of an infant does not entirely depend on the care of the midwife."*

Our diet, he tells us, contains too much meat, too little vegetable. We should eat in moderation, taking a good breakfast but a light supper.

> *"It were well for mankind if cookery, as an art, were entirely prohibited."*

Alcohol (in moderation of course) is best prepared in the home; apparently commercial liquors were often laced with opiates.

On infection he is quite clear:

> *"If the patient has the small pox or any other infectious disease there is no doubt that the doctor's clothes, hands etc. will carry away some of the infection and if he goes directly to visit another patient without washing his hands, changing or being exposed to the open air ... is it any wonder that he should carry the disease along with him."*

Take away only the antiseptic properties of fresh air and he is a hundred years ahead of his time. On 'telling the patient' he is equally forthright:

> *"A sensible patient had better hear what the doctor says than learn it from the disconsolate looks, the watery eyes and the broken whispers of those about him."*

His phraseology is arresting:

> *"It is nature alone that cures wounds. All that we do is to remove obstacles ..."*
>
> *All nervous patients, without exception, are afflicted with wind."*

I have dwelt at some length upon Buchan not only because his book was one of the most popular books of its type ever written, but because it is essentially modern in approach and refreshingly free from self advertisement.

The triumphs of the nineteenth century were, in general, not those of internal medicine but of surgery – but these are triumphs seen in retrospect. Our domestic medicine authors were more cautious – anyway most of them were physicians. In 1844 for example:

> *"An operation for the removal of an ulcerated cancer of the breast is now never performed by men of judgement and very rarely recom-*

*mended by them in unbroken cancer except in the earlier stage. Persons at a distance from London will do well to note this as it is by no means uncommon for a quite different advice to be given in the country."*

thus wrote Thomas Graham whose opinions (and prejudices) may well last into the next millennium. A manual of Domestic Medicine published in 1882 by a "group of physicians of the principal London hospitals" is clearly intended to tell an individual reader how to look after himself, rather than to appeal, as did many of the earlier books to clergymen or ladies bountiful who could then practice their amateur art upon their hapless tenantry. The authors pay a gracious compliment to the discoverers of anaesthetics:

*"The discovery of the value of the subcutaneous injection of morphine, of local anaesthesia by freezing .... and of general anaesthetics ... may rank amongst the proudest triumphs of this or any other age."*

The comment on the other major surgical advance of the century is also revealing. Lister had come to London only five years before and his ideas were slow to gain acceptance yet in domestic practice:

*"The employment (of disinfectants) is generally an indication that the nursing has been at fault. There are cases, of course, of foul discharges from the body which require the employment of chemical deodorizers but even these cases,* **since the introduction of the antiseptic method of treating wounds** *are now of far less frequent occurrence than formerly."*

The somewhat pompous style of the English physicians is altogether blown away by the breeziness of their American counterparts. If I quote from the learned Edward B. Foote M.D. (may be consulted daily excepting Sundays in the English or German language at 120 Lexington Avenue, corner of East 42nd St., New York) it is because his work is so delightfully illustrated and his conclusions so unequivocal. In the late nineteenth century there was considerable interest in physiognomy and phrenology and Foote links this with the Galenical ideas of the temperaments. The illustrations leave little room for diagnostic doubt and show in addition how it is possible to foretell marriage failure from a simple study of physiognomy; marriage indeed was a subject upon which Foote held strong opinions:

*"There should be a board of physiologists, well versed in the science of temperaments, physiognomy phrenology etc. whose functions shall consist in the power to examine into the mental and physical characteristics of candidates for matrimony, to grant or refuse licences ... and to grant divorces to those who are miserably mated in wedlock."*

If this book seems to us to contain opinionated nonsense it nevertheless had sales of over a million; I have found five copies in Cornwall alone. It deserves its influence if only for passages like this:

"Natures calls Imperative;– *is it indeed a disagreeable task, one we are ashamed of, to dispose of the useless portions of the liquids and solids we have put into our mouths? May we not better teach our children to be ashamed of besmearing their mouths with vile tobacco and loading their breath with the vapours of unwholesome drinks? May we not better place a gate at the door wherein so much that is injurious enters than stop up the outlets from which many things purer depart?"*

The book is wide ranging – it even includes a section on 'Rapacious Doctors' – and it contains a long and detailed account of sexual and marriage customs around the world. Here a certain coyness comes in and one senses that there are limits beyond which even a medical author may not stray. My favourite illustration demonstrates those limits better than I can. Finally before we cross the Atlantic once again let us leave Dr. Foote with the last word on sharing a bed:

*"Is it not a little damaging to the romantic element of a refined nature to meet the companion you love in a nightcap and nightgown at night and then to behold the whole night gear thoroughly messed on arising in the morning. Where there is one 'sleeping beauty' there are a hundred persons, who, in their slumbers look like facial contortionists. Throw a glance at the sleepers in public conveyances if you don't want to look at home ..."*

ENCEPHALIC TEMPERAMENT.

**Fig 3**

Could he be ...

LYMPHATIC TEMPERAMENT.

**Fig 4**

... happy with her?

LOCAL INADAPTATION.

*Note.*—With the greatest effort on the part of the author and engraver, it has been found difficult to present these illustrations in a way to avoid offence to those who are not individually or professionally interested. The course suggested in the *Note* on the opposite page has therefore been chosen, and they will be supplied to the barren or to physicians free of charge.

**Fig 5**

When we come to the present century we see a further change of emphasis. At the turn of the century one finds multi-volume prestige publications such as Cassell's Family Physician whose readers are anxious to know where to convalesce, which are the best British and continental watering places. We explore the delicate social quandary – does the sick nurse eat with the servants or with the family? We are told how to instruct the maid to prepare the sick room or to furnish a room prior to a surgical operation. The problem of transporting the invalid thereafter seems easily solved:

> *"... there is nothing better than a hammock. If a real hammock is not to be obtained there is generally but little trouble in getting one made. There is no jarring in a hammock and it can be fastened up in an ordinary omnibus or saloon railway carriage without any difficulty." My illustration shows also a "simple alternative which does not form an inconvenient addition to one's luggage."*

**Fig 6**

"A simple alternative"

After the First World War more 'popular' books are produced and the publishing houses and newspapers seem to have vied with each other to create the definitive text. This was an era of therapeutic optimism, of a dawning realisation that the Nation's health was in some way the responsibility of the Nation's Government. It was a time when improvements in hygiene and industrial health were at last beginning to reverse the tide of disease. It was a time when the languid and effete hothouse flowers of Mr. Chavasse's Victorian drawing rooms had been hardened off into the Woman's League of Health and Beauty. The "Golden Health Library" under the editorship of Sir Arbuthnot Lane is an exuberant celebration of Health – the illustration I have selected says almost all that is necessary. The whole tone is one of optimism; death rates from infectious diseases are falling (this is 1930), surgery is burgeoning, a clean air act is transforming our cities. Cancer? "Cancer *never attacks a healthy organ.* (For prevention) the obvious course is to keep fit by eating natural foods, by paying most careful attention to the sufficiently frequent and regular action of the bowels ..." and so on. With so many citadels of disease falling who can doubt but that the remainder will soon surrender? Meantime here is the recipe for healthy living, the Regimen Sanitatis Britanniae.

**Fig 7**

Says it all

## Figures and Tables

**Fig 1 f1:**
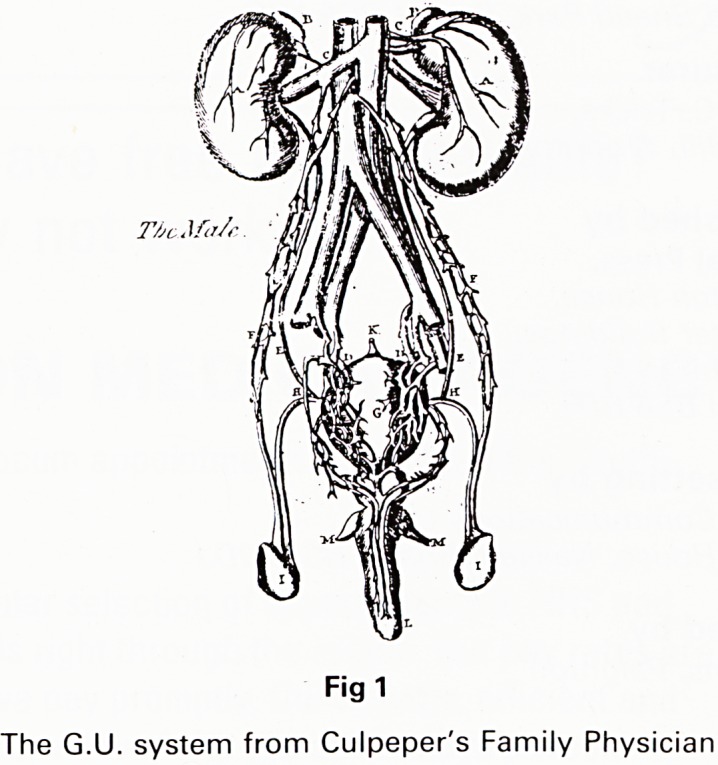


**Fig 2 f2:**
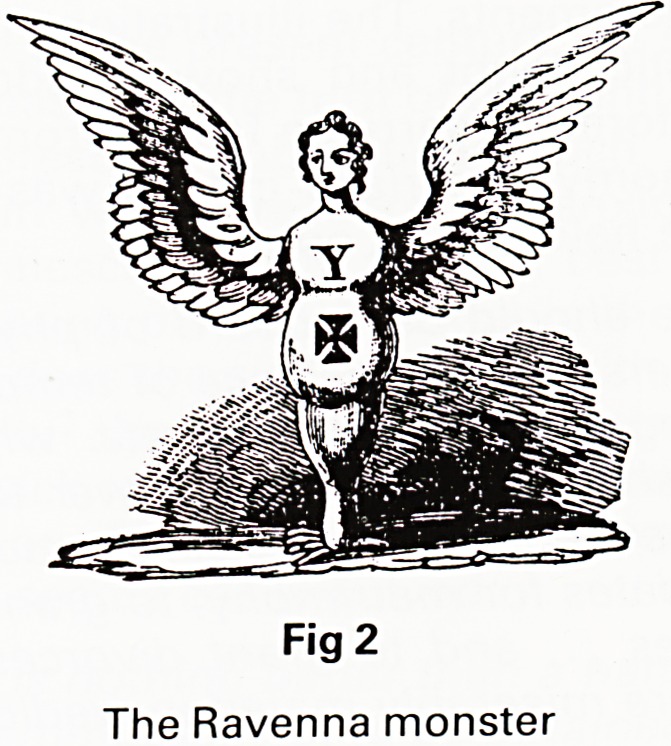


**Fig 3 f3:**
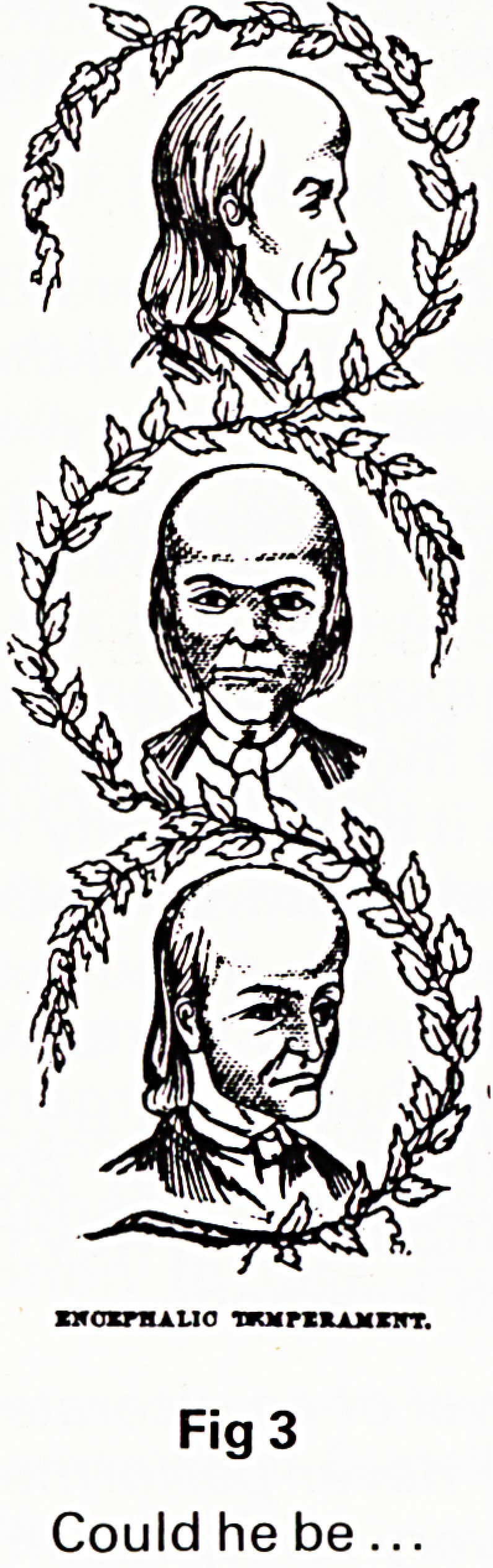


**Fig 4 f4:**
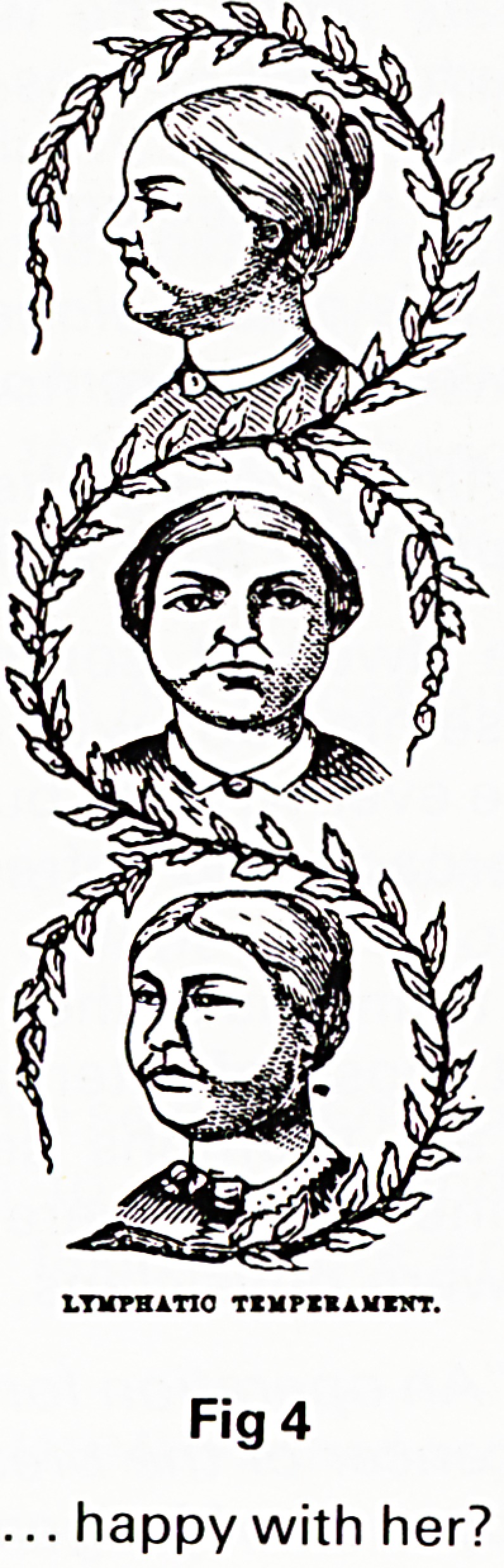


**Fig 5 f5:**
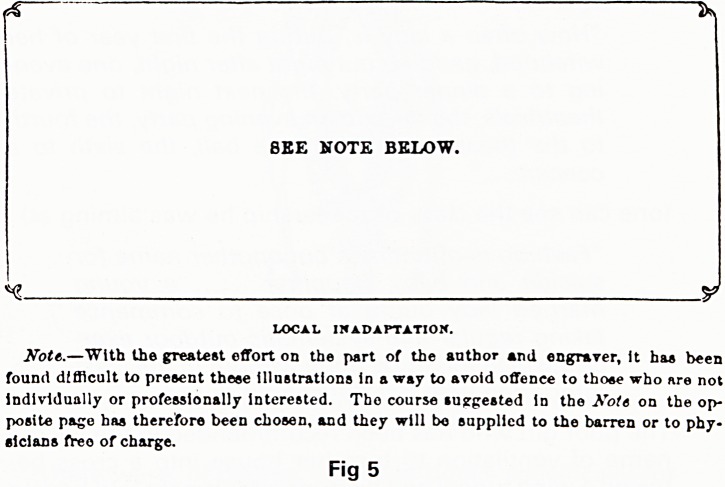


**Fig 6 f6:**
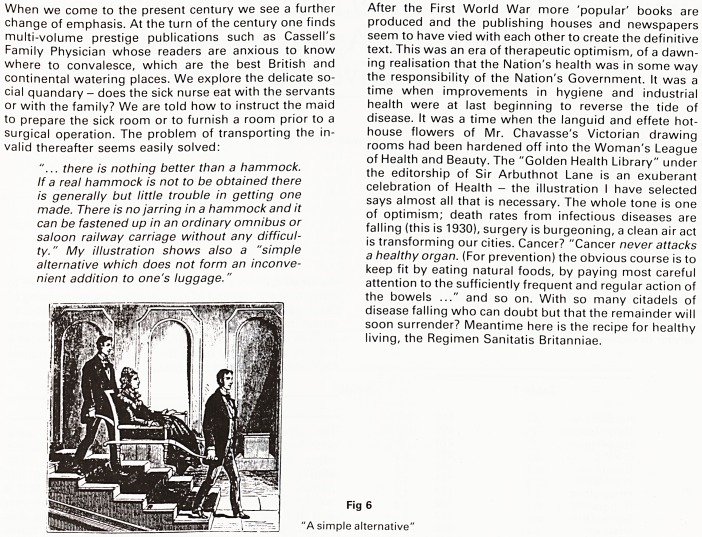


**Fig 7 f7:**